# Impact of postanesthesia care unit delirium on self-reported cognitive function and perceived health status: a prospective observational cohort study

**DOI:** 10.1007/s11136-022-03087-1

**Published:** 2022-01-27

**Authors:** Elena Kainz, Karin Stuff, Ursula Kahl, Christian Wiessner, Yuanyuan Yu, Franziska von Breunig, Rainer Nitzschke, Alexander Haese, Markus Graefen, Marlene Fischer

**Affiliations:** 1 Department of Anesthesiology, University Medical Center Hamburg-Eppendorf, Martinistrasse 52, 20246 Hamburg, Germany; 2 Institute of Medical Biometry and Epidemiology, University Medical Center Hamburg-Eppendorf, Martinistrasse 52, 20246 Hamburg, Germany; 3 Martini-Klinik Prostate Cancer Center, University Medical Center Hamburg-Eppendorf, Martinistrasse 52, 20246 Hamburg, Germany; 4 Department of Intensive Care Medicine, University Medical Center Hamburg-Eppendorf, Martinistrasse 52, 20246 Hamburg, Germany

**Keywords:** Anesthesia recovery period, Delirium, Postoperative cognitive complications, Cognitive dysfunction, Quality of life, Prostatectomy, Perceived health status

## Abstract

**Purpose:**

The objective of this study was to determine the influence of postanesthesia care unit (PACU) delirium on self-reported cognitive function and perceived health status 3 months after surgery.

**Methods:**

This prospective observational cohort study was performed in a PACU at a high-volume prostate cancer center. We used a convenience sample of patients > 60 years undergoing elective radical prostatectomy. Patients with a history of cerebrovascular or neurodegenerative disease were excluded. Fifteen, 30, 45, and 60 following extubation, patients were screened for signs of delirium with the Confusion Assessment Method for the Intensive Care Unit. Three months after surgery self-reported cognitive function was assessed with the Cognitive Failures Questionnaire, and health status was evaluated with the 36-item Short-Form Health Survey (SF-36).

**Results:**

Signs of PACU delirium were present in 32.4% (*n* = 72/222) of patients, and 80.2% (*n* = 178/222) completed the 3-month follow-up. The presence of PACU delirium signs was not significantly associated with self-reported cognitive failures (B = 0.60, 95% CI: −1.72; 2.92, *p* = 0.61) or SF-36 physical component scores (B = 0.19, 95% CI: 0.02; 0.36, *p* = 0.03) or SF-36 mental component scores (B =  −0.03, 95% CI: −0.18, 0.11, *p* = 0.66) 3 months after radical prostatectomy.

**Conclusions:**

In a cohort of educated, highly functioning, elderly male patients who were assessed immediately after surgery and at a 3-month follow-up, we found no association between PACU delirium and self-reported cognitive failures or perceived health status, which implies that PACU delirium may be an event of limited duration and impact.

**Trial registration:**

The study was registered at ClinicalTrials.gov (Identifier: NCT04168268, Date of registration: November 19, 2019).

**Supplementary Information:**

The online version contains supplementary material available at 10.1007/s11136-022-03087-1.

## Introduction

Perioperative neurocognitive disorders, including postoperative delirium (POD), are common severe complications after surgery and anesthesia that particularly affect elderly patients [1, 2]. Delirium occurs in up to 50% of patients within one week of major non-cardiac surgery [3, 4]. According to the fifth edition of the *Diagnostic and Statistical Manual of Mental Disorders* (DSM-5), delirium is defined as an acute onset and typically reversible syndrome of diffuse brain dysfunction [5]. It is characterized by disturbances in attention and awareness and cognitive impairment with fluctuating severity that are not better explained by pre-existing neurocognitive disorders or severe reduction of arousal [5]. Postoperative delirium can be further classified based on the time of onset in relation to surgical intervention and anesthesia. Delirium signs in the postanesthesia care unit (PACU) develop after emergence from anesthesia during the immediate postoperative period [6]. Postanesthesia care unit delirium has been observed in up to 45% of patients after elective surgery and is a likely predictor of subsequent POD [6].

Postoperative delirium has been linked to adverse outcomes, including increased length of hospital and intensive care unit stay, higher institutionalization rates, rising healthcare costs, and increased mortality [7, 8]. Furthermore, there is growing evidence that POD is associated with reduced health status after surgery, persistent cognitive impairment, and dementia [9–13].

While evidence of the long-term consequences of POD is mounting, it remains unclear whether PACU delirium is associated with similar effects. It is still unclear, whether PACU delirium is a transient phenomenon or an enduring medical condition with relevant adverse impact. Recent findings suggest that even short episodes of PACU delirium are associated with poor in-hospital outcomes, including impaired cognitive function compared with preoperative cognitive performance [14]. Data on the impact of PACU delirium after hospital discharge are limited. Cognitive decline, in particular, has been shown to affect health perception [15].

We hypothesized that PACU delirium would be associated with higher self-reported cognitive failures and reduced perceived health status 3 months after radical prostatectomy.

## Methods

### Ethical approval

The study protocol was approved by the local ethics committee at the Hamburg Chamber of Physicians (protocol No. 4782/Prof. Dr. M. Carstensen/02.09.2014). Written informed consent was obtained from all patients prior to participation. The study was registered at ClinicalTrials.gov (Identifier: NCT04168268).

### Study design, setting, and participants

This prospective, observational study was performed at a high-volume prostate cancer center in Northern Germany between November 2017 and October 2018. Male patients aged over 60 years who were admitted to the PACU after elective open radical retropubic prostatectomy (ORP) or robot-assisted radical prostatectomy (RARP) were eligible for study participation. Patients were required to be fluent in German to answer all questionnaires. The exclusion criteria were pre-existing neurological or cerebrovascular conditions, a history of dementia, or mild cognitive impairment. We chose a convenience sample of patients, who were admitted between Mondays and Thursdays on the day before surgery. Patients, who declined to participate, who were absent from the ward or who were prescheduled for postoperative transfer to the intensive care unit (ICU) were not eligible.

### Preoperative psychometric evaluation

Preoperative baseline assessments were performed by one trained anesthesiologist following a standardized protocol. We used the Mini-Mental Status Examination (MMSE) to screen for dementia or mild cognitive impairment [16] and the Patient Health Questionnaire-9 (PHQ-9) to assess depressive symptoms [17]. We conducted baseline PHQ-9 screening, as symptoms of depression should be considered when interpreting delirium, subjective cognitive complaints, and quality of life. Depression has previously been proven to be a risk factor for POD, especially in older patients [18, 19], and it is associated with patient-reported cognitive failures [20, 21] and quality of life [22, 23]. The Cognitive Failures Questionnaire (CFQ) was used to evaluate the type and frequency of self-reported cognitive failures in everyday life [24]. It assesses errors in three areas: life perception, memory, and motor function. The CFQ consists of 25 items. The ratings (0–4) of the individual items are summed to obtain a total score between 0 and 100, with a higher score indicating more self-reported cognitive failures [24]. The Cognitive Reserve Index (CRI) questionnaire, which addresses education, profession, and leisure time activities as the three main sources of cognitive reserve, was administered to measure the quantity of cognitive reserve accumulated through a lifespan [25]. A complete list of assessments performed throughout the perioperative period is presented in Online Resource 1.

### Assessment of PACU delirium

To screen for signs of delirium, a trained examiner interviewed all participants 15, 30, 45, and 60 min after arrival at the PACU. At each of these time points, the patients’ level of arousal was assessed using the Richmond Agitation and Sedation Scale (RASS) [26]. Patients with an RASS score of −4 (no response to voice but movement or eye opening in response to physical stimulation) or −5 (no response to voice or physical stimulation) were ineligible for delirium assessment and were re-evaluated 15 min later. If the patients showed a reasonable reaction (i.e., an RASS score between −3 and + 4), the Confusion Assessment Method for the Intensive Care Unit (CAM-ICU) was performed. The CAM-ICU allows for delirium screening in less than 3 min [27, 28]. It comprises four domains that assess the following delirium criteria: (1) an acute onset of mental status change or a fluctuating course; (2) inattention; (3) disorganized thinking, and (4) an altered level of consciousness. A positive CAM-ICU score indicates signs of an acute change in mental status or fluctuating course (feature 1) accompanied by inattention (feature 2), and either disorganized thinking or an altered level of consciousness (feature 3 or 4) [27]. The CAM-ICU is an effective, validated and highly specific tool for delirium diagnosis and can be performed easily at the bedside [27]. Importantly, the CAM-ICU has a higher specificity than sensitivity for the detection of delirium during the recovery period [29]. Postanesthesia care unit delirium was defined as a positive CAM-ICU score together with an RASS score greater than or equal to −3 (any response to verbal stimulation). Participants were also asked to rate their pain perception on a numerical scale between 0 and 10 at the time of delirium screening. All delirium assessments in the PACU were performed by two trained examiners, who were both members of the research team (E.K., M.F.). Repeated assessments in one study participant were performed by one examiner.

### Three-month follow-up

Three months after surgery, we assessed physical and mental health status using the 36-item Short-Form Health Survey (SF-36). Self-reported cognitive failures in everyday life were evaluated with the CFQ. All study participants received questionnaires by mail. The SF-36 is a validated questionnaire that assesses eight health concepts. The eight domains can be categorized into two summary measures: physical and mental health component scores [30]. Higher scores indicate a better self-reported health status [31]. Raw SF-36 data from the completed questionnaires were transferred to an online analysis tool (Hogrefe Testsystem 5, Testzentrale, Göttingen, Germany) for further processing according to a predefined algorithm.

### Surgical and anesthesiologic management

The choice of surgical technique (ORP or RARP) was based on individual risk factors, surgical considerations, oncological factors, and patient preference. General anesthesia was administered according to our institutional standard operating procedures. Sufentanil (0.3–0.7 μg/kg) and propofol (2–3 mg/kg) were used for induction of general anesthesia, followed by neuromuscular blockade with rocuronium (0.6 mg/kg) to facilitate endotracheal intubation. A gastric tube was inserted in all patients and prophylactic antiemetic medication was administered preoperatively (dexamethasone 4 mg). Intraoperatively, rocuronium was titrated under the guidance of neuromuscular blockade monitoring (train-of-four, TOF-Watch Organon; IntelliVue NMT module, Philips GmbH, Hamburg, Germany), and anesthesia depth was monitored using a bispectral index monitor (BIS™, Medtronic GmbH, Meerbusch, Germany). Sevoflurane-sufentanil was used for anesthesia maintenance to achieve an end-tidal sevoflurane concentration of 2.0 vol% (MAC 0.8–1.2). Normothermia was maintained using a forced-air warming system throughout the entire procedure. Patients who underwent RARP received peritoneal insufflation with carbon dioxide and were positioned at a 45-degree head-down tilt. Postoperative pain management included non-opioid medication (metamizole 1000 mg/100 ml) 30 min before emergence and every 4–6 h thereafter. During the PACU stay, piritramide 3.75–7.5 mg was administered intravenously when pain scores exceeded 3. Subsequent PACU management in all patients included frequent control of wound drains, postoperative urine output, blood gas analyses, and pain management as described above.

### Statistical analysis

Details on data collection are presented in Online Resource 2. Continuous data are presented as medians with interquartile ranges (IQR), and categorical data are presented as frequencies with percentages. For group comparisons (no PACU delirium vs. PACU delirium), the Mann–Whitney U-test (continuous variables), Chi-square test, or Fisher’s exact test (categorical variables) were used as appropriate.

The association between PACU delirium and our outcome measures was assessed with three separate path diagrams, one for each outcome. For path analyses, we selected clinically relevant variables that did not fulfill the criteria for collinearity (Online Resource 2). The path diagrams were formed by clinical considerations about the dependencies of the variables. An important advantage of path diagrams over conventional regression methods is that mediating effects can be evaluated. Of main interest were the paths from PACU delirium to the outcomes, and the paths that lead into PACU delirium. Thereby, the effects of variables on either PACU delirium or the outcome measure can be distinguished in one model.

Additional mediation analyses were performed for associations of PACU delirium duration on each of our outcome measures (CFQ, SF-36 physical, and SF-36 mental component score). The following subgroups were formed to define and differentiate PACU delirium duration: (1) patients who were delirious at least at one of assessment points 2–4 (30, 45 or 60 min after arriving in the PACU; n=35), and (2) patients with delirium 15 mins after arriving in the PACU only (n=37).

For path analyses, a p-value of less than 0.017 was considered statistically significant (Bonferroni-corrected for multiplicity). We used IBM SPSS Statistics 24 (IBM Corporation, Armonk, New York), and STATA 16 (STATA Corporation, College Station, Texas) for statistical analyses. This manuscript adheres to the STROBE reporting guidelines for observational studies.

## Results

### Study population

**Fig. 1:**
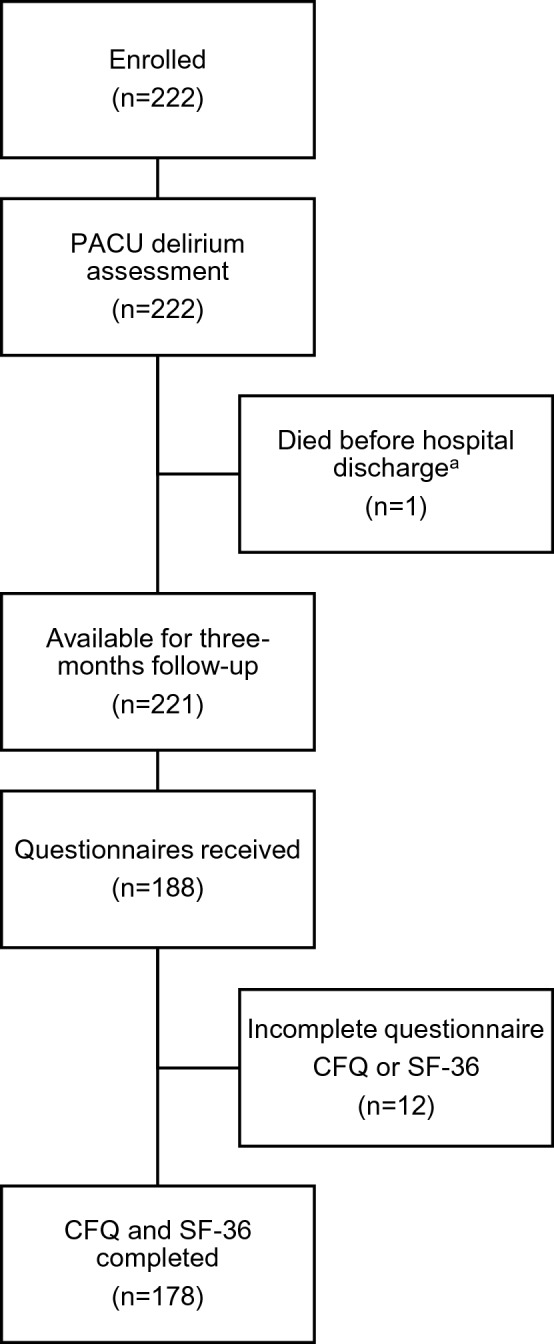
Flow of participants. *PACU* Postanesthesia care unit, *CFQ* Cognitive Failures Questionnaire, *SF-36* 36-item Short Form Health Survey. ^a^Refractory cardiogenic shock after myocardial infarction

A total of 222 patients were enrolled and assessed for the development of PACU delirium. One patient died during the early postoperative period. The remaining patients were discharged to home after a median length of hospital stay of 6 days (IQR: 6–7). Two hundred and twenty-one subjects were available for follow-up after 3 months. Questionnaires for both endpoints, CFQ and SF-36, were returned by 178 patients (80.5% of survivors) and were included in the statistical analyses (Fig. 1). Detailed descriptive data stratified by response status are presented in Online Resource 3.

### Patients’ characteristics

Patient’s characteristics divided by signs of PACU delirium are shown in Table 1. The median age of patients with PACU delirium was higher than that of patients without PACU delirium. The majority of participants had low perioperative risk and were classified as category I or II in the American Society of Anesthesiologists physical status classification system (89.6%, *n* = 199/222). There was no relevant imbalance of baseline psychometric properties between groups. Detailed information on psychometric scores at baseline is provided in Table 1.

**Table 1:** Patients’ characteristics, baseline psychometric assessment, and patient-reported outcomes at 3 months stratified by delirium status

	No PACU delirium (*n* = 150)	PACU delirium (*n* = 72)	*p*
Age (years)	66 (63–70)	69 (65–72)	0.014
BMI (kg/m^2^)	25.4 (24.2–27.5)	26.3 (24.8–28.7)	0.133
*ASA physical status classification system*			0.802
ASA I	13 (8.70)	8 (11.10)	
ASA II	122 (81.30)	56 (77.80)	
ASA III	15 (10.00)	8 (11.10)	
*Pre-existing conditions*			
Obesity (BMI ≥ 30)	14 (9.3)	9 (12.5)	0.469
Arterial hypertension	86 (57.3)	38 (52.8)	0.522
Coronary heart disease	17 (11.3)	4 (5.6)	0.223
Diabetes/prediabetes	11 (7.3)	8 (11.1)	0.346
Dyslipoproteinemia	43 (28.7)	15 (20.8)	0.214
COPD	3 (2.0)	2 (2.8)	0.660
Current smoking status	13 (8.7)	4 (5.6)	0.591
*Baseline psychometric assessment*			
MMSE	30 (28–30)	29 (29–30)	0.490
PHQ-9	2 (1–4)	2 (1–5)	0.368
CRI education	113 (103–129)	112 (101–127)	0.482
CRI working activity	125 (115–133)	117 (107–131)	0.021
CRI leisure time	127 (116–139)	124 (110–138)	0.420
CRI total	131 (119–141)	125 (115–139)	0.144
CFQ preoperative sum score	15 (10–21)	17 (12–23)	0.179
CFQ preoperative forgetfulness	8 (6–11)	9 (7–11)	0.415
CFQ preoperative distractibility	5 (2–7)	5 (3–8)	0.165
CFQ preoperative false triggering	2 (1–4)	2 (1–5)	0.385
*PACU delirium and patient-reported outcomes at 3 months*			
*Cognitive Failures Questionnaire (n=178)*			
CFQ postoperative sum score	14 (6–22)	18 (8–24)	0.217
CFQ postoperative forgetfulness	8 (5–10)	9 (4–12)	0.305
CFQ postoperative distractibility	4 (1–7)	5 (1–8)	0.165
CFQ postoperative false triggering	2 (1–5)	4 (1–6)	0.088
*SF-36 (n=178)*			
SF-36 physical component score	52.6 (47.3–55.5)	50.6 (42.3–54.9)	0.045
SF-36 mental component score	55.6 (49.9–57.8)	55.1 (49–57.7)	0.927
*SF-36 subcategories*			
Physical functioning	90 (85–95)	90 (65–95)	0.005
Role physical	100 (50–100)	75 (25–100)	0.212
Bodily pain	100 (74–100)	100 (74–100)	0.399
General health	72 (62–82)	67 (57–82)	0.342
Vitality	70 (60–80)	65 (55–75)	0.229
Social functioning	100 (75–100)	88 (75–100)	0.192
Role emotional	100 (100–100)	100 (100–100)	0.772
Mental health	84 (72–92)	80 (72–88)	0.095
Change in health state	3 (3–4)	3 (2–3)	0.011

*PACU* Postanesthesia care unit, *ASA* American Society of Anesthesiologists, *BMI* Body mass index, *COPD* Chronic obstructive pulmonary disease, *MMSE* MiniMental State Examination, *PHQ-9* Patient Health Questionnaire-9, *CRI* Cognitive Reserve Index, *CFQ* Cognitive Failures Questionnaire, *SF-36* 36-item Short Form Health survey. Continuous variables are presented as median with interquartile range. Categorial variables are shown as absolute numbers with percentages

**Fig. 2:**
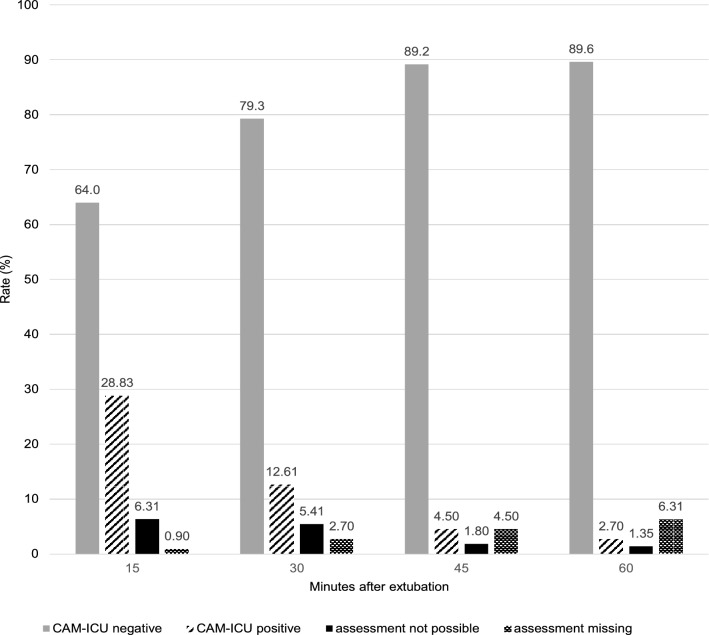
Incidence of PACU delirium during the first hour after extubation. *PACU* Postanesthesia care unit, *CAM-ICU* Confusion Assessment Method for the Intensive Care Unit. Data are presented as relative numbers. Assessment not possible: application of the CAM-ICU was not possible due to agitation or lethargy/no response. Assessment missing refers to patients with a score < −3 on the Richmond Assessment Agitation Scale or patients, who had urological physical and/or sonographic examination at the time of delirium screening: 15 min: *n* = 2; 30 min: *n* = 6; 45 min: *n* = 10; 60 min: = 14

Delirium signs were present in 32.4% of patients (*n* = 72/222). The incidence was highest 15 min after extubation and decreased over time, with the lowest delirium rates occurring 60 min after extubation. Details of the incidence of PACU delirium during the first hour after extubation are presented in Fig. 2. Hypoactive delirium was the predominant type of delirium in our study population. The majority of patients diagnosed with PACU delirium showed hypoactive features, whereas only one patient was in a hyperactive state 15 min upon arrival at the PACU.

### Anesthesiologic management and surgery

Robot-assisted radical prostatectomy was performed in 51.8% (*n* = 115/222) and ORP in 48.2% (*n* = 107/222) of study participants. The surgical approach, RARP vs. ORP, did not differ significantly between patients with and without delirium (*p* = 0.840). The durations of both surgery and anesthesia were significantly longer in patients with PACU delirium (Table 2). There was no significant difference in the estimated blood loss or the amount of fluids administered between patients with and without delirium. The length of PACU stay and the dosage of analgesic and antiemetic drugs administered during the recovery period did not differ significantly between patients with and without PACU delirium. Table 2 shows the details of anesthesiologic and surgical management stratified by delirium status. The laboratory parameters are listed in Online Resource 4.

**Table 2:** Perioperative characteristics by delirium status

	No PACU-deliriumz *n* = 150	PACU-delirium *n* = 72	*p*
Type of surgery			0.840
*RARP*	77 (51.30)	38 (52.80)	
*ORP*	73 (48.70)	34 (47.20)	
Duration of surgery (min)	165 (150–190)	185 (165–200)	0.001
Duration of anesthesia (min)	240 (220–265)	255 (233–285)	0.001
Sufentanil (total amount, µg)	85 (75–98)	90 (80–100)	0.073
Noradrenaline (µg per kg per min)	0.06 (0.04–0.09)	0.05 (0.03–0.07)	0.025
Estimated blood loss (ml)	425 (200–700)	500 (300–800)	0.187
Fluids (total amount, ml)	2500 (2000–3000)	2500 (2000–3000)	0.780
Atropin (administered)	16 (10.7)	12 (16.7)	0.207
Length of PACU stay (min)	170 (135–210)	175 (153–210)	0.225
Piritramide (total amount, mg)	7.5 (0–7.5)	3.8 (0–7.5)	0.096
Pain scores^a^			
*NRS-1*	2 (1–3)	1 (0–3)	<0.001
*NRS-2*	3 (1–3)	2 (0–3)	0.033
*NRS-3*	3 (2–4)	3 (1–4)	0.442
*NRS-4*	3 (2–4)	3 (1–5)	0.333
Antiemetic drugs^b^			0.308
*None*	141 (94.00)	65 (90.30)	
*Dimenhydrinate 62 mg*	0 (0.00)	1 (1.40)	
*Ondansetron 4 mg*	8 (5.30)	6 (8.30)	
*Ondansetron + dimenhydrinate*	1 (0.70)	0 (0.00)	
Vasopressor support^b,c^	10 (6.70)	4 (5.60)	1.000
Lactate (mmol per l)^d^	1.1 (0.8–1.4)	1 (0.8–1.4)	0.930
SpO_2_^b^	98 (97–99)	98.3 (97.5–99)	0.759
Supplemental oxygen (ml per h)^b^	4 (4–4)	4 (4–4)	0.157
Length of hospital stay (days)	6 (6–7)	6 (6–7)	0.993
Clavien-Dindo classification^e^			0.691^f^
*No postoperative complications*	135 (90.0)	66 (91.7)	
*Grade I*	4 (2.7)	2 (2.8)	
*Grade II*	6 (4.0)	2 (2.8)	
*Grade III*	0 (0.0)	1 (1.4)	
*Grade IIIa*	4 (2.7)	1 (1.4)	
*Grade IIIb*	0 (0.0)	0 (0.0)	
*Grade IV*	0 (0.0)	0 (0.0)	
*Grade IVa*	0 (0.0)	0 (0.0)	
*Grade IVb*	0 (0.0)	0 (0.0)	
*Grade V*	1 (0.7)	0 (0.0)	
UICC			0.104
*II (T2b or T2c)*	77 (51.3)	27 (37.5)	
*III (T3)*	54 (36.0)	30 (41.7)	
*IV (T4 or N1 or M1)*	19 (12.7)	15 (20.8)	
Additive therapy			0.016
*None*	127 (84.7)	51 (70.8)	
*Adjuvant ADT + radiotherapy*^g^	23 (15.3)	21 (29.2)	

*ADT* Androgen deprivation treatment, *PACU* Postanesthesia care unit, *RARP* Robot-assisted radical prostatectomy, *ORP* Open radical retropubic prostatectomy, *NRS* Numeric rating scale, *SpO2* Peripheral oxygen saturation, *UICC* Union internationale contre le cancer. Continuous variables are presented as median with interquartile range. Categorical variables are presented as absolute and relative numbers. ^a^Reported pain perception on a numerical rating scale (0–10) at the four time points of delirium assessment. ^b^During the PACU stay. ^c^Noradrenaline was administered continuously to maintain a mean arterial blood pressure ≥ 65 mmHg. ^d^Arterial (RARP) or venous (ORP) blood gas analysis during the PACU stay. ^e^At discharge from hospital. ^f^Chi-square test for the comparison of postoperative complications (“yes” vs. “no”) between patients with and without PACU delirium. ^g^Based on multidisciplinary board decisions adjuvant radiotherapy was initiated 3 months after surgery

### PACU delirium and patient-reported outcomes at 3 months

Cognitive failures at the 3-month follow-up were not significantly different between patients with (18 [IQR 8–24]) and without delirium (14 [IQR 6–22], *p* = 0.217). Patients diagnosed with PACU delirium scored lower in the physical component of the SF-36 after 3 months compared to patients without PACU delirium (50.6 [IQR 42.3–54.9] vs. 52.6 [IQR 47.3–55.5], *p* = 0.04). The mental health component scores did not differ between both groups (55.1 [IQR 49–57.7] vs. 55.6 [IQR 49.9–57.8], *p* = 0.927). Details of patient-reported outcomes at 3 months are presented in Table 1.

**Fig. 3:**
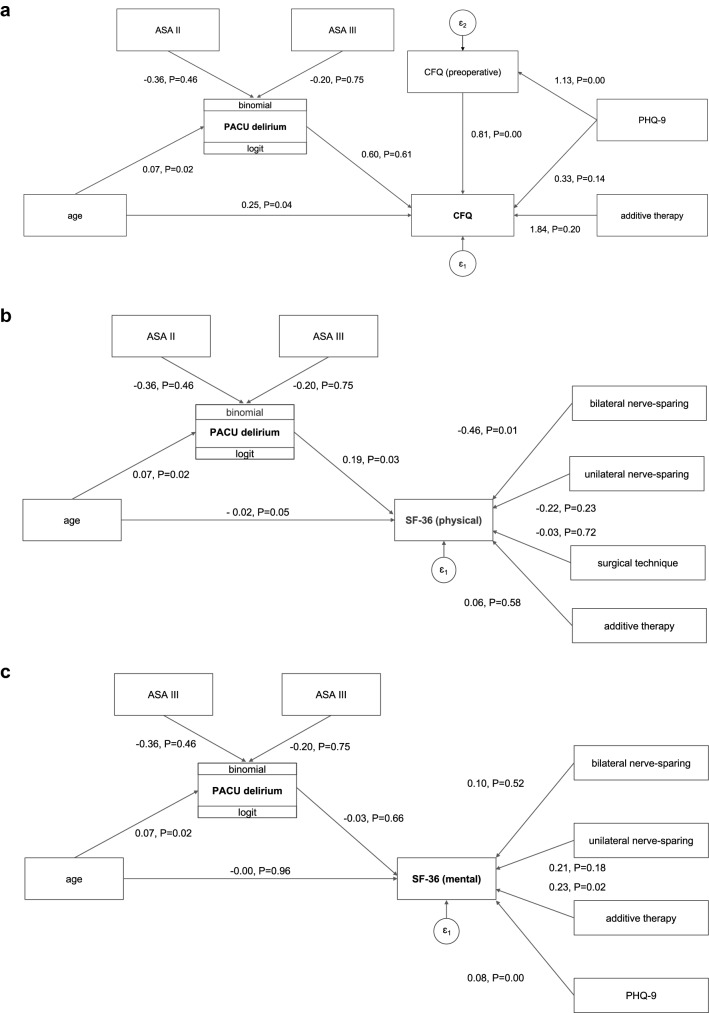
Path diagrams for cognitive failures (**a**), SF-36 physical component score (**b**), and SF-36 mental component score (**c**). Postanesthesia care unit (PACU) delirium was included as an endogenous variable to assess a potential mediation effect on patient-reported outcomes. *ASA* American Society of Anesthesiologists, *CFQ* Cognitive Failures Questionnaire, *PHQ-9* Patient Health Questionnaire, *SF-36* Short Form Health Survey

There was no significant association between PACU delirium and self-reported cognitive failures after controlling for age, PHQ-9, the CFQ baseline score, and additive therapy in our study population (Fig. 3a, Table 3). There was a statistically significant association between a higher preoperative CFQ sum score and more self-reported cognitive failures 3 months after surgery (*p* < 0.01). The effect of the PHQ-9 score on cognitive failures at 3 months was fully mediated by the number of preoperative cognitive failures (*p* < 0.01).

**Table 3:** Influence of PACU delirium on cognitive failures, SF-36 physical health and SF-36 mental health after 3 months

		B	95%CI	*P*
*Cognitive failures*				
	Age → PACU delirium	0.07	0.01; 0.14	0.02
	ASA II → PACU delirium	−0.36	−1.31; 0.59	0.46
	ASA III → PACU delirium	−0.20	−1.45; 1.04	0.75
	PACU delirium → CFQ	0.60	−1.72; 2.92	0.61
	Age → CFQ	0.25	0.01; 0.50	0.04
	CFQ preoperative sum score → CFQ	0.81	0.66; 0.95	<0.01
	PHQ-9 → CFQ	0.33	−0.11; 0.77	0.14
	Additive therapy → CFQ	1.84	−1.00; 4.68	0.20
	PHQ-9 → CFQ preoperative sum score	1.13	0.76; 1.49	<0.01
*SF-36 physical component score*				
	Age → PACU delirium	0.07	0.01; 0.14	0.02
	ASA II → PACU delirium	−0.36	−1.31; 0.59	0.46
	ASA III → PACU delirium	−0.20	−1.45; 1.04	0.75
	PACU delirium → SF-36 physical component score	0.19	0.02; 0.36	0.03
	Age → SF-36 physical component score	−0.02	−0.04; −0.00	0.05
	Bilateral nerve-sparing → SF-36 physical component score	−0.46	−0.80; −0.11	0.01
	Unilateral nerve-sparing → SF-36 physical component score	−0.22	−0.59; 0.14	0.23
	Surgical technique → SF-36 physical component score	−0.03	−0.19; 0.13	0.72
	Additive therapy → SF-36 physical component score	0.06	−0.16; 0.29	0.58
*SF-36 mental component score*				
	Age → PACU delirium	0.07	0.01; 0.14	0.02
	ASA II → PACU delirium	−0.36	−1.31; 0.59	0.46
	ASA III → PACU delirium	−0.20	−1.45; 1.04	0.75
	PACU delirium → SF-36 mental component score	−0.03	−0.18; 0.11	0.66
	Age → SF-36 mental component score	−0.00	−0.02; 0.02	0.96
	Bilateral nerve-sparing → SF-36 mental component score	0.10	−0.20; 0.40	0.52
	Unilateral nerve-sparing → SF-36 mental component score	0.21	−0.10; 0.53	0.18
	Additive therapy → SF-36 mental component score	0.23	0.04; 0.43	0.02
	PHQ-9 → SF-36 mental component score	0.08	0.06; 0.11	<0.01

Path analyses for mediation effects of PACU delirium in 178 patients, who had completed the 3-month follow-up. *ASA* American Society of Anesthesiologists, *CFQ* Cognitive Failures Questionnaire, *CI* Confidence interval, *CRI* Cognitive Reserve Index, *PACU* Postanesthesia care unit, *PHQ-9* Patient Health Questionnaire-9, *SF-36* 36-item Short Form Health Survey

No significant association was found between PACU delirium and the SF-36 physical component score after controlling for age, nerve-sparing resection, type of surgery, and additive therapy. The direct effect of age on the physical component score was partially mediated by PACU delirium, although not reaching statistical significance (Fig. 3b, Table 3).

There was no significant association between PACU delirium and the SF-36 mental component score after controlling for age, nerve-sparing resection, additive therapy, and PHQ-9 score (Fig. 3c, Table 3). The PHQ-9 score at baseline was significantly associated with the mental component score at 3 months (*p* < 0.01). There was a statistical trend for the effect of additive therapy on the mental component score (*p* = 0.02).

In subgroup mediation analyses there was no statistically significant association of PACU delirium duration on cognitive failures and SF-36 physical and mental component scores. Details on the subgroup analysis are presented in Online Resource 5.

## Discussion

In the present study PACU delirium was not associated with self-reported cognitive function, physical or mental SF-36 component scores 3 months after surgery. Results of previous studies indicate that the risk of poor long-term outcomes increases progressively with the duration of delirium episodes [14, 32, 33]. One prospective observational study showed that multiple days of delirium in a mixed medical-surgical ICU were associated with self-reported cognitive failures after one year. However, one single day with delirium signs was not associated with more cognitive failures compared to no occurrence of delirium [34]. These findings are in line with a longitudinal study that evaluated cognitive and executive functions in survivors of critical illness at 3 and 12 months after discharge [33]. The authors found significant cognitive impairment and executive dysfunction, both of which depended on the duration of delirium [33]. Neufeld and colleagues showed an independent association of early PACU delirium with impaired short-term cognition at hospital discharge compared with preoperative assessment [14]. In a secondary analysis, they observed a potential dose–response relationship between delirium duration and negative outcomes. Patients with POD beyond the PACU stay showed more severe impairment of cognitive function than patients without delirium signs or those with delirium signs during the PACU stay only. The same study cohort was also assessed for long-term impact of PACU delirium 18 months after surgery. The authors did not observe adverse effects on mortality, cognitive or functional impairment, or health care utilization [34]. These results are in line with our finding of a lack of association between PACU delirium and adverse outcomes at 3 months. Therefore, it is conceivable that short PACU delirium episodes cause only a transient impairment of cognitive function.

We did not find a significant association between the number of positive delirium assessments and cognitive failures or SF-36 physical and mental component scores in the subgroup analyses. Our findings are in contrast to the results of Neufeld and coworkers described above [14]. It is important to note, however, that delirium assessment started around 45 min after operating room exit and was continued until day five after surgery, while delirium screening in our study population was performed between 15 min and 60 min after admission to the PACU. Since our data cover a more limited time interval, it is possible that minor differences of duration were not sufficient to demonstrate a significant dose–response relationship of PACU delirium duration on patient-reported outcomes in our study.

Cognitive impairment has been shown to affect physical health and to promote physical frailty [15]. Persistent cognitive decline as a consequence of delirium may affect health perception [15, 35]. Therefore, we hypothesized that, similar to POD, PACU delirium would be associated with reduced self-reported cognitive function and an impairment of mental and physical health. In contrast to our hypothesis, we did not observe adverse effects of PACU delirium on either physical or mental SF-36 component scores. Observational studies have reported reduced health-related quality of life after delirium episodes [13, 36]. Abelha and colleagues found an association between POD after major non-cardiac and non-neurological surgeries and reduced health-related quality of life after 6 months in a surgical ICU population [13]. Domains of physical functioning, vitality, and social functioning, which relate to both the physical and mental health aspects of the SF-36, were particularly affected [13]. Although POD appears to have adverse effects on self-reported health status, we did not observe similar negative findings for PACU delirium. Considerable differences in study populations, timing of delirium assessments, and uncertain onset of delirium episodes may have led to these contradictory findings. Our results might be another indication of a limited impact of short delirium episodes diagnosed in the PACU.

We found higher preoperative CFQ scores to be associated with more cognitive failures after 3 months. This finding might be explained by the stability of the CFQ over long periods, which Broadbent and colleagues described as similar to that of traditional measures of trait rather than state [24].

Higher preoperative depression scores were associated with more preoperative cognitive complaints. Thus, our findings reflect the impact of depressive symptoms on subjective cognitive failures, which has been consistently reported in previous research [20, 21]. Furthermore, we observed an adverse impact of higher PHQ-9 scores on the mental component score. It seems plausible that symptoms associated with depression affect the mental health summary measure, which is mainly determined by the SF-36 domains “social functioning”, “mental health”, and “role emotional”. This is in accordance with previous results on the adverse effects of depression on health-related quality of life and surgical and functional outcome in oncologic patients [23, 37, 38].

Nerve-sparing radical prostatectomy has been shown to be associated with improved patient-reported outcomes compared to non-nerve-sparing procedures [22, 39]. This is in line with our findings of an association between bilateral nerve-sparing surgery and higher physical component scores at 3 months.

### Strengths and limitations

Several caveats should be considered when interpreting the results of this prospective observational study. We used the CAM-ICU, which has a higher specificity (98%) than sensitivity (28%) for delirium diagnosis in the PACU setting compared to a formal psychiatric evaluation based on DSM criteria [29]. Thus, the low sensitivity of the CAM-ICU when conducted in the recovery room may have led to underdiagnosis of PACU delirium. However, in contrast to evaluation based on DSM criteria, the CAM-ICU can be performed easily and rapidly at the bedside and is, therefore, more likely to be adopted into daily routines, making it a suitable tool for PACU delirium screening. At the time of data collection for our study, there was no screening tool available that was sensitive, specific to PACU delirium, and timesaving to use. In the meantime, Hight and colleagues have published a modified and extended version of the CAM-ICU assessment tool for delirium screening in the recovery room (CAM-PACU) [40]. The CAM-PACU score may have higher sensitivity as it includes additional criteria and may, therefore, be considered for PACU delirium screening in future studies [40].

Cognitive assessment of our study population was based on the CFQ, which is the most widely used tool for measuring patient-reported cognitive failures [41]. Importantly, self-reported cognitive function may not necessarily correlate with objective assessment [41]. We may have missed subtle changes in postoperative cognitive performance that might have been detected with an objective neuropsychological assessment. However, the two methods represent different but equally valuable approaches to evaluating cognitive function [41]. Thus, the use of subjective measures in conjunction with an objective neuropsychological assessment should be considered in future studies that investigate the impact of PACU delirium. Importantly, self-reported cognitive function represents one essential patient-centered outcome, which is why we chose this endpoint for our trial.

We did not assess delirium signs beyond 1 h after arrival in the PACU and follow-up was performed only once at 3 months. Therefore, we cannot comment on the continued trajectories of delirium signs and possible subsequent effects on cognitive function and perceived health status during the early postoperative period. Of all study participants, 32.4% screened positive for delirium at one or more time point during the first hour, with the highest delirium rates recorded early after extubation. The fact that the number of patients with delirium features decreased with time suggests that signs of PACU delirium resolve rapidly.

The timing of the follow-up in our study may have biased the results regarding the impact of PACU delirium on cognitive functions. By assessing delirium during the PACU period only, we may have missed single patients with persisting delirium beyond the recovery period. Inouye and colleagues published a model showing a biphasic course of cognitive trajectories after POD, characterized by an acute peak of cognitive dysfunction, subsequent recovery, and a gradual decline throughout 36 months. At 2 months, there was no significant difference between delirious and non-delirious patients [9]. The postulated recovery phase includes our follow-up time point (3 months after surgery). Therefore, it is possible that our finding of no adverse effects of PACU delirium was due to the dynamic course of cognitive impairment.

Our study population was homogeneous with relatively highly educated, high-functioning, elderly male patients without pre-existing cognitive impairment and with low perioperative risk undergoing elective radical prostatectomy. Thus, the generalizability of our findings might be limited and should be confirmed in more diverse samples.

The homogeneity of our study population, all of whom underwent highly standardized surgical interventions, anesthesia, and perioperative care within a single clinical setting, is also a remarkable strength of our study design as it ensures almost identical timing and ambient conditions of PACU delirium screening of all patients.

The analysis of self-reported health status in addition to cognitive outcomes adds to important current knowledge on the intermediate- and long-term impact of PACU delirium.

Furthermore, our data illustrate the reproducibility and feasibility of PACU delirium screening during the first hour after extubation. Since PACU delirium may be a predictor of subsequent POD and its associated adverse outcomes [6], we strongly support systematic delirium screening in the PACU setting to identify patients who might be particulary susceptible to prolonged episodes of delirium. In a previous observational trial, our research group found an incidence of PACU delirium of 48% in patients over 60 years of age. Interestingly, the majority of patients had received midazolam for premedication, which might explain the higher incidence of PACU delirium [42].

## Conclusion

In a cohort of educated, highly functioning, elderly male patients, we found no significant association between PACU delirium and self-reported cognitive failures, physical or mental health status 3 months after elective surgery. Our data suggest that PACU delirium may be a transient condition with decreasing impact simultaneous to the waning of delirium signs. However, our findings should be interpreted with caution due to their limited generalizability. Whether PACU delirium, similar to later POD, is associated with adverse patient-reported outcomes warrants investigation in future trials including more diverse patient populations.

## Supplementary Information

Below is the link to the electronic supplementary material.

Supplementary file1—Assessments throughout the study period. (PDF 75 kb)

Supplementary file2Data collection and variable selection for path analyses. (PDF 68 kb)

Supplementary file3—Baseline demographic, clinical, and psychometric characteristics stratified by response status. (PDF 91 kb)

Supplementary file4—Laboratory parameters. (PDF 105 kb)

Supplementary file5—Subgroup analyses of PACU delirium duration on cognitive failures and SF-36 physical and mental component scores at three months. (DOCX 17 kb)

## Data Availability

The datasets used and/or analyzed during the current study are available from the corresponding author on reasonable request.
