# Supplementary material for: Impact of postanesthesia care unit delirium on self-reported cognitive function and perceived health status: a prospective observational cohort study
Source: Qual Life Res. 2022 Jan 27;31(8):2397–410. doi: 10.1007/s11136-022-03087-1 (PMC9250471; doi:10.1007/s11136-022-03087-1)
Supplement: Supplementary file 1 — Supplementary file1—Assessments throughout the study period. (PDF 75 kb) [file 11136_2022_3087_MOESM1_ESM.pdf]

Title: Impact of postanesthesia care unit delirium on self-reported cognitive function and perceived health status: a prospective observational cohort study

Journal name: Quality of Life Research

Author names: Elena Kainz, Karin Stuff, Ursula Kahl, Christian Wiessner, Yuanyuan Yu, Franziska von Breunig, Rainer Nitzschke, Alexander Haese, Markus Graefen, Marlene Fischer.

Corresponding author: Marlene Fischer, University Medical Center Hamburg-Eppendorf, Department of Anesthesiology, Department of Intensive Care Medicine, Martinistrasse 52, 20246 Hamburg, Germany. Email: mar.fischer@uke.de

Supplementary file 1: Assessments throughout the study period.

Preoperative assessments and baseline scores
- MMSE: cognitive impairment
- PHQ-9: depressive symptoms
- CFQ: cognitive failures
- CRI: cognitive reserve

Screening for PACU delirium
- RASS: agitation and sedation
- CAM-ICU: PACU delirium screening
- Numerical Rating Scale: pain

3-month follow-up
- CFQ: cognitive failures
- SF-36: perceived health status

Supplementary file 1: Assessments throughout the study period. MMSE: Mini-Mental Status Examination. PHQ-9: Patient Health Questionnaire-9. CFQ: Cognitive Failures Questionnaire. CRI: Cognitive Reserve Index. PACU: postanesthesia care unit. RASS: Richmond Agitation and Sedation Scale. SF-36: 36-item Short Form Health Survey.
