# Supplementary material for: Impact of postanesthesia care unit delirium on self-reported cognitive function and perceived health status: a prospective observational cohort study
Source: Qual Life Res. 2022 Jan 27;31(8):2397–410. doi: 10.1007/s11136-022-03087-1 (PMC9250471; doi:10.1007/s11136-022-03087-1)
Supplement: Supplementary file 2 — Supplementary file2Data collection and variable selection for path analyses. (PDF 68 kb) [file 11136_2022_3087_MOESM2_ESM.pdf]

Title: Impact of postanesthesia care unit delirium on self-reported cognitive function and perceived health status: a prospective observational cohort study

Journal name: Quality of Life Research

Author names: Elena Kainz, Karin Stuff, Ursula Kahl, Christian Wiessner, Yuanyuan Yu, Franziska von Breunig, Rainer Nitzschke, Alexander Haese, Markus Graefen, Marlene Fischer.

Corresponding author: Marlene Fischer, University Medical Center Hamburg-Eppendorf, Department of Anesthesiology, Department of Intensive Care Medicine, Martinistrasse 52, 20246 Hamburg, Germany. Email: mar.fischer@uke.de

Supplementary file 2: Data collection and variable selection for path analyses.

Data collection

Baseline scores, demographic characteristics, and information on comorbidities were obtained during the pre-anesthesia visit. Variables related to anesthesia and surgery were retrieved from the anesthesia protocol, pre- and postoperative laboratory parameters, and uropathological features were collected from electronic patient charts.

Variable selection for path analyses

The path diagram for cognitive failures included “age”, “American Society of Anesthesiologists physical status”, “PHQ-9 score”, and “additive therapy” as exogenous variables and “PACU delirium” and “CFQ preoperative sum score” as endogenous variables. The path diagram for the SF-36 physical component score included “age”, “American Society of Anesthesiologists physical status”, “nerve sparing RP” (bilateral, unilateral, none), “type of surgery”, and “additive therapy” as exogenous and “PACU delirium” as endogenous variables. The path diagram for the SF-36 mental component score included “age”, “American Society of

Anesthesiologists physical status", "nerve sparing RP" (bilateral, unilateral, none), "additive therapy", and "PHQ-9 score" as exogenous and "PACU delirium" as endogenous variables. Patients who were lost to follow-up at 3 months were omitted from path analyses. Both SF-36 component scores were transformed prior to analysis because they were left-skewed. The following formulas were used for transformation: "63-ln(SF-36 physical)" and "68-ln(SF-36 physical".
