# Supplementary material for: Impact of postanesthesia care unit delirium on self-reported cognitive function and perceived health status: a prospective observational cohort study
Source: Qual Life Res. 2022 Jan 27;31(8):2397–410. doi: 10.1007/s11136-022-03087-1 (PMC9250471; doi:10.1007/s11136-022-03087-1)
Supplement: Supplementary file 3 — Supplementary file3—Baseline demographic, clinical, and psychometric characteristics stratified by response status. (PDF 91 kb) [file 11136_2022_3087_MOESM3_ESM.pdf]

Title: Impact of postanesthesia care unit delirium on self-reported cognitive function and perceived health status: a prospective observational cohort study

Journal name: Quality of Life Research

Author names: Elena Kainz, Karin Stuff, Ursula Kahl, Christian Wiessner, Yuanyuan Yu, Franziska von Breunig, Rainer Nitzschke, Alexander Haese, Markus Graefen, Marlene Fischer.

Corresponding author: Marlene Fischer, University Medical Center Hamburg-Eppendorf, Department of Anesthesiology, Department of Intensive Care Medicine, Martinistrasse 52, 20246 Hamburg, Germany. Email: mar.fischer@uke.de

Supplementary file 3: Baseline demographic, clinical, and psychometric characteristics stratified by response status.

	**Non-responder n=32**	**CFQ or SF-36 n=12**	**CFQ and SF-36 n=178**
PACU delirium	8 (25.00)	2 (16.70)	62 (34.80)
Age (years)	68 (63-70)	70 (69-73)	67 (64-71)
BMI (kg/m^2)	25 (24.4-28.2)	25.3 (24.3-25.8)	25.8 (24.4-27.8)
ASA physical status classification system			
ASA I	3 (9.40)	0 (0.00)	18 (10.10)
ASA II	25 (78.10)	11 (91.70)	142 (79.80)
ASA III	4 (12.50)	1 (8.30)	18 (10.10)
Obesity (BMI ≥ 30)	2 (6.30)	1 (8.30)	20 (11.20)
Arterial hypertension	15 (46.90)	7 (58.30)	102 (57.30)
Coronary heart disease	3 (9.40)	1 (8.30)	17 (9.60)
Diabetes/prediabetes	3 (9.40)	0 (0.00)	16 (9.00)
Dyslipoproteinemia	8 (25.00)	5 (41.70)	45 (25.30)
COPD	1 (3.10)	0 (0.00)	4 (2.20)
Current smoking status	3 (9.40)	0 (0.00)	14 (7.90)
RARP	19 (59.40)	5 (41.70)	91 (51.10)
ORP	13 (40.60)	7 (58.30)	87 (48.90)
Duration of surgery (min)	175 (153-195)	175 (155-203)	175 (150-195)
Duration of anesthesia (min)	240 (225-278)	250 (230-278)	245 (225-270)
Sufentanil (total amount, µg)	83 (74-93)	83 (70-98)	90 (75-100)

Noradrenaline (µg per kg per min)	0.06 (0.03-0.07)	0.05 (0.04-0.09)	0.06 (0.03-0.09)
Estimated blood loss (ml)	450 (225-650)	575 (300-850)	450 (250-750)
Fluids (ml per min)	10.24 (8.7-11.32)	10.47 (9.81-12.15)	10.2 (8.33-12.1)
Length of PACU stay (min)	180 (143-205)	178 (125-193)	170 (140-210)
Piritramide (total amount, mg)	5.6 (0-7.5)	7.5 (0-7.5)	5.5 (0-7.5)
Atropin (administered)	1 (3.10)	2 (16.70)	25 (14.00)
MMSE	30 (29-30)	30 (29-30)	30 (29-30)
PHQ-9	2 (1-7)	1 (0-2)	2 (1-4)
CRI education	116 (102-125)	116 (104-130)	113 (102-128)
CRI working activity	123 (109-138)	129 (125-131)	123 (110-132)
CRI leisure time	119 (109-138)	131 (118-142)	126 (114-139)
CRI total	129 (110-142)	130 (125-142)	128 (117-141)
CFQ preoperative sum score	16 (10-24)	12 (10-15)	16 (10-22)
CFQ preoperative forgetfulness	8 (6-11)	8 (7-9)	9 (6-11)
CFQ preoperative distractibility	6 (1-5)	4 (2-5)	5 (2-7)
CFQ preoperative false triggering	3 (1-5)	2 (0-3)	2 (1-4)

Supplementary file 3. PACU: postanesthesia care unit. ASA: American Society of Anesthesiologists. COPD: chronic obstructive pulmonary disease. RARP: robot-assisted radical prostatectomy. ORP: open radical retropubic prostatectomy. BMI: body mass index. MMSE: Mini-Mental Status Examination. PHQ-9: Patient Health Questionnaire-9. CRI: Cognitive Reserve Index. CFQ: Cognitive Failures Questionnaire. Continuous variables are presented as median with interquartile range. Categorical variables are presented as absolute and relative number.
