# Supplementary material for: Impact of postanesthesia care unit delirium on self-reported cognitive function and perceived health status: a prospective observational cohort study
Source: Qual Life Res. 2022 Jan 27;31(8):2397–410. doi: 10.1007/s11136-022-03087-1 (PMC9250471; doi:10.1007/s11136-022-03087-1)
Supplement: Supplementary file 4 — Supplementary file4—Laboratory parameters. (PDF 105 kb) [file 11136_2022_3087_MOESM4_ESM.pdf]

Title: Impact of postanesthesia care unit delirium on self-reported cognitive function and perceived health status: a prospective observational cohort study

Journal name: Quality of Life Research

Author names: Elena Kainz, Karin Stuff, Ursula Kahl, Christian Wiessner, Yuanyuan Yu, Franziska von Breunig, Rainer Nitzschke, Alexander Haese, Markus Graefen, Marlene Fischer.

Corresponding author: Marlene Fischer, University Medical Center Hamburg-Eppendorf, Department of Anesthesiology, Department of Intensive Care Medicine, Martinistrasse 52, 20246 Hamburg, Germany. Email: mar.fischer@uke.de

Supplementary file 4: Laboratory parameters.

	**no PACU-delirium n=150**	**PACU-delirium n=72**	***p***
Hemoglobin$_{\textbf{preoperative}}$ (g/dl)	14.3 (13.7-15.1)	14.4 (13.5-15.1)	0.941
Hemoglobin$_{\textbf{postoperative}}$ (g/dl)	11.4 (10.8-12.3)	11.5 (10.8-12.3)	0.972
Δhemoglobin (g/dl)	2.7 (2.3-3.3)	2.7 (2.3-3.5)	0.800
Sodium$_{\textbf{postoperative}}$ (mmol/l)	140 (139-141)	140 (139-142)	0.327
Potassium$_{\textbf{postoperative}}$ (mmol/l)	4.1 (3.9-4.2)	4 (3.8-4.2)	0.165
Creatinine$_{\textbf{preoperative}}$ (mg/dl)	1 (0.91-1.1)	0.94 (0.85-1)	0.003
Creatinine$_{\textbf{postoperative}}$ (mg/dl)	0.89 (0.81-0.99)	0.84 (0.75-0.94)	0.004
Δcreatinine (mg/dl)	0.1 (0.03-0.17)	0.11 (0.02-0.16)	0.782
C-reactive protein$_{\textbf{postoperative}}$ (mg/l)	42 (29-55)	43 (33-57)	0.433
White blood cells$_{\textbf{postoperative}}$ (G/l)	9.4 (7.4-11.3)	101 (8-12.6)	0.118

Supplementary file 4. PACU: postanesthesia care unit. Variables are presented as median with interquartile range. Δ refers to the difference between pre- and postoperative serum levels.
