# Supplementary material for: Impact of postanesthesia care unit delirium on self-reported cognitive function and perceived health status: a prospective observational cohort study
Source: Qual Life Res. 2022 Jan 27;31(8):2397–410. doi: 10.1007/s11136-022-03087-1 (PMC9250471; doi:10.1007/s11136-022-03087-1)
Supplement: Supplementary file 5 — Supplementary file5—Subgroup analyses of PACU delirium duration on cognitive failures and SF-36 physical and mental component scores at three months. (DOCX 17 kb) [file 11136_2022_3087_MOESM5_ESM.pdf]

Title: Impact of postanesthesia care unit delirium on self-reported cognitive function and perceived health status: a prospective observational cohort study

Journal name: Quality of Life Research

Author names: Elena Kainz, Karin Stuff, Ursula Kahl, Christian Wiessner, Yuanyuan Yu, Franziska von Breunig, Rainer Nitzschke, Alexander Haese, Markus Graefen, Marlene Fischer.

Corresponding author: Marlene Fischer, University Medical Center Hamburg-Eppendorf, Department of Anesthesiology, Department of Intensive Care Medicine, Martinistrasse 52, 20246 Hamburg, Germany. Email: mar.fischer@uke.de

Supplementary file 5: Subgroup analyses of PACU delirium duration on cognitive failures and SF-36 physical and mental component scores at 3 months

	**Parameter Estimate**	**95% CI**	***p***
**CFQ**			
Age → PACU duration	0.16	[0.04; 0.28]	0.011
ASA II → PACU duration	1.31	[-0.45; 3.07]	0.144
ASA III → PACU duration	3.06	[0.38; 5.75]	0.025
PACU duration → CFQ	1.13	[-2.57; 4.83]	0.550
Age → CFQ	-0.05	[-0.45; 0.34]	0.788
CFQ (preoperative) → CFQ	1.04	[0.80; 1.28]	<0.001
PHQ-9 → CFQ	0.18	[-0.48; 0.85]	0.587
Additive Therapy → CFQ	1.79	[-2.23; 5.81]	0.383
PHQ-9 → CFQ (preoperative)	0.93	[0.40; 1.46]	0.001
**SF-36 physical component score**			
Age → PACU duration	0.16	[0.04; 0.28]	0.011
ASA II → PACU duration	1.31	[-0.45; 3.07]	0.144
ASA III → PACU duration	3.06	[0.38; 5.75]	0.025
PACU duration → SF-36 physical	0.21	[-0.07; 0.48]	0.136
Age → SF-36 physical	-0.03	[-0.07; -0.00]	0.026
Bilateral nerve-sparing → SF-36 physical	-0.37	[-0.88; 0.14]	0.151
Unilateral nerve-sparing → SF-36 physical	0.19	[-0.36; 0.75]	0.499
Surgical technique → SF-36 physical	-0.22	[-0.50; 0.05]	0.106
Additive Therapy → SF-36 physical	0.27	[-0.07; 0.61]	0.122
**SF-36 mental component score**			

Age → PACU duration	0.16	[0.04; 0.28]	0.011
ASA II → PACU duration	1.31	[-0.45; 3.07]	0.144
ASA III → PACU duration	3.06	[0.38; 5.75]	0.025
PACU duration → SF-36 mental	0.02	[-0.22; 0.25]	0.899
Age → SF-36 mental	0.01	[-0.02; 0.03]	0.578
Bilateral nerve-sparing → SF-36 mental	0.05	[-0.39; 0.48]	0.836
Unilateral nerve-sparing → SF-36 mental	-0.06	[-0.53; 0.41]	0.800
Additive Therapy → SF-36 mental	0.18	[-0.10; 0.46]	0.215
PHQ-9 → SF-36 mental	0.08	[0.04; 0.12]	<0.001

Supplementary file 5. Associations of PACU delirium duration on outcome measures. Following subgroups were defined to define delirium duration: (1) patients who were delirious at least at one of delirium assessment points 2-4 (30, 45 or 60 minutes after arriving in the PACU; n=35) and (2) patients with delirium only fifteen minutes after arriving in the PACU (n=37). CFQ: Cognitive Failures Questionnaire. PACU: postanesthesia care unit. PHQ-9: Patient Health Questionnaire-9. ASA: American Society of Anesthesiologists. SF-36: 36-item Short Form Health survey.
